# Functional Self-Awareness and Metacontrol for Underwater Robot Autonomy

**DOI:** 10.3390/s21041210

**Published:** 2021-02-09

**Authors:** Esther Aguado, Zorana Milosevic, Carlos Hernández, Ricardo Sanz, Mario Garzon, Darko Bozhinoski, Claudio Rossi

**Affiliations:** 1 Centre for Automation and Robotics UPM-CSIC, Universidad Politécnica de Madrid, 28006 Madrid, Spain; Zorana.Milosevic@upm.es (Z.M.); Ricardo.Sanz@upm.es (R.S.); Claudio.Rossi@upm.es (C.R.); 2 Resources Computing International Ltd, Matlock DE4 5JA, UK; 3 Cognitive Robotics, Delft University of Technology, 2628 CD Delft, The Netherlands; C.H.Corbato@tudelft.nl (C.H.); M.A.GarzonOviedo@tudelft.nl (M.G.); D.Bozhinoski@tudelft.nl (D.B.)

**Keywords:** autonomy, resilience, self-awareness, metacontrol, rendundancy, ontology

## Abstract

Autonomous systems are expected to maintain a dependable operation without human intervention. They are intended to fulfill the mission for which they were deployed, *properly handling the disturbances* that may affect them. Underwater robots, such as the UX-1 mine explorer developed in the UNEXMIN project, are paradigmatic examples of this need. Underwater robots are affected by both external and internal disturbances that hamper their capability for autonomous operation. Long-term autonomy requires not only the capability of perceiving and properly acting in open environments but also a sufficient degree of robustness and resilience so as to maintain and recover the operational functionality of the system when disturbed by unexpected events. In this article, we analyze the operational conditions for autonomous underwater robots with a special emphasis on the UX-1 miner explorer. We then describe a knowledge-based self-awareness and metacontrol subsystem that enables the autonomous reconfiguration of the robot subsystems to keep mission-oriented capability. This resilience augmenting solution is based on the deep modeling of the functional architecture of the autonomous robot in combination with ontological reasoning to allow self-diagnosis and reconfiguration during operation. This mechanism can transparently use robot functional redundancy to ensure mission satisfaction, even in the presence of faults.



**Contents**
 1      Introduction · · · · · · · · · · · · · · · · · · · · · · · · · · · · · · · · · · · · · · · · · · · · · · · ·2 2      State of the Art · · · · · · · · · · · · · · · · · · · · · · · · · · · · · · · · · · · · · · · · · · · · · ·3
2.1      Flexible Approach to Robust Autonomy in UUVs · · · · · · · · · · · · 3
2.2      Resilient Robotics · · · · · · · · · · · · · · · · · · · · · · · · · · · · · · · · · · · · · ·4
2.3      Fault-Tolerance and Adaptation · · · · · · · · · · · · · · · · · · · · · · · · · ·5
2.4      Self-X for Robots · · · · · · · · · · · · · · · · · · · · · · · · · · · · · · · · · · · · · · ·6 3      The UX-1 Robot: A Flooded Mine Explorer · · · · · · · · · · · · · · · · · · · · · · ·6 4      A Metacontrol Architecture Using Self-Knowledge · · · · · · · · · · · · · · · ·9
4.1      TOMASys: Metacontrol Framework · · · · · · · · · · · · · · · · · · · · · · ·10
4.2      Ontological Reasoning and Metacontrol · · · · · · · · · · · · · · · · · · · ·11 5      UX-1 Self-Model and Metacontrol · · · · · · · · · · · · · · · · · · · · · · · · · · · · · ·12
5.1      UX-1 Ontological Model · · · · · · · · · · · · · · · · · · · · · · · · · · · · · · · · ·12
5.2      Ontological Reasoning for UX-1 · · · · · · · · · · · · · · · · · · · · · · · · · ·13
5.3      Reconfiguration in UX-1 · · · · · · · · · · · · · · · · · · · · · · · · · · · · · · · · ·15 6      Experimental Results · · · · · · · · · · · · · · · · · · · · · · · · · · · · · · · · · · · · · · · ·17 7      Discussion · · · · · · · · · · · · · · · · · · · · · · · · · · · · · · · · · · · · · · · · · · · · · · · · ·24
7.1      Assessment of Present State · · · · · · · · · · · · · · · · · · · · · · · · · · · · ·25
7.2      Future Work · · · · · · · · · · · · · · · · · · · · · · · · · · · · · · · · · · · · · · · · · ·25 8      Concluding Remarks · · · · · · · · · · · · · · · · · · · · · · · · · · · · · · · · · · · · · · · · ·25 References · · · · · · · · · · · · · · · · · · · · · · · · · · · · · · · · · · · · · · · · · · · · · · · · · · · · · ·26


## 1. Introduction

Unmanned Underwater Vehicles (UUV), also called Autonomous Underwater Robots (AUR) and Autonomous Underwater Vehicles (AUV), perform a variety of operations in situations that are far from being fully controlled. They perform in unstructured and hazardous environments with high uncertainty and, at the same time, they shall operate robustly because the possibility of human intervention to solve occurring problems is minimal in deep waters.

The most widespread applications of these robots are exploratory in nature, moving in partially unknown environments to perform reconnaissance, inspection or mapping tasks of both the environment and target objects of interest. These are common in the scientific and military domains, as well as in industries such as mining, oil and gas that commonly use UUVs in the exploitation activities of underwater resources or operation and maintenance of submerged elements.

These robots share control architectures with other autonomous mobile robots like Unmanned Ground Vehicles (UGV) or Unmanned Aerial Vehicles (UAV), because the essence of their activities are the same: motion, sensing, localization, mapping, navigation, propulsion or control. However, due to the specifics of each environment, the concrete system architectures shall put some emphasis on specific aspects that are of special relevance in each robot, environment and task (e.g., wind disturbance in UAVs, moving pedestrians in UGVs and pressure handling in UUVs). Of all the three environments, the underwater one is of particular difficulty because the medium—water—poses major difficulties especially concerning visibility (both optical and radio) and physical affectation (esp. pressure and humidity). This derives in special requirements for UUVs to handle these conditions.

Robot autonomy requires capabilities for performing a task without help, but it also requires capabilities for enduring disturbances when performing a task. Disturbances are common trade in the underwater environment and may come both from the environment and from the robot itself. UUVs, as any technical system, are susceptible to failure in any subsystem or component. All the subsystems, —propulsion, communications, sensors, power supply, mechanical elements, among several others—are prone to failure due to the harsh conditions where they operate. This implies that these robots, when deployed in real environments, have *very concrete operational requirements* that specifically address these extreme operational conditions and failure possibilities. Besides the usual operational requirements that cover the mission specification and the infrastructure needed for the operation; environmental conditions such as pressure, temperature, humidity, or radiation must be taken into account. Also, redundancy and fault-handling techniques are commonly set as operational requirements to extend the life cycle of the artifact. In field applications of UUVs it is crucial to deploy specific system recovery functionalities, capable of keeping the robot operating in the presence of disturbances that may produce task disruption, component faults or even physical damage. Mission continuity is critical to complete the task –fully or partially– or, at least, to be able to return to the robot base.

The UX-1 UUV [1], is an autonomous explorer for submerged mines [2]. It serves as a suitable platform to deploy and test self-awareness and self-control concepts targeted at augmenting its autonomy. In the UX-1 we transcend the classic fault-tolerance capabilities to deploy general self-awareness and reconfiguration capabilities to increase the reliability of the system by improving its adaptive resilience. The objective of our research is producing a robot able to recover after damage, using and adapting redundant or alternative parts of the system to keep performing the same task in complete or diminished form. This approach, departs from the concrete, ad-hoc component redundancies that are commonly used, to a generic, knowledge-based, mission-level strategy for system adaptation.

The core capability of this fault-tolerant controllers is achieved through the introduction of a knowledge-based *metacontroller*. Just as a traditional controller has to maintain a variable value close to a certain reference set-point, the metacontroller targets as reference the *specified functionality of the system*. In case the system deviates from the functional reference, a system reconfiguration is in need. A reasoner exploits a metamodel-driven knowledge base, specific for the robot and its mission, to produce a new suitable configuration.

However, several difficulties must be addressed to reach a system capable to evolve according to necessities during the lifetime of the application. This may imply a human-in-the-loop architecture in which engineer knowledge is used to evolve the operative agent. As the system is operating, metrics such as accuracy and efficiency need to be monitored to detect to what extent goals are being reached. Moreover, the reliability of the run-time knowledge must be evaluated. The implementation of these capabilities represents a challenge to engineers. This challenge, however, addresses the problem of increasing the autonomy levels of robotic systems.

This paper presents four contributions to augment robots’ autonomy levels. (1) The use of self-awareness and diagnosis techniques to evaluate the robot performance both at module level and at system level, (2) the use of ontological reasoning to trace fault sources, (3) the production of system design alternatives in real-time, and (4) a proof-of-concept of these techniques as applied to the UX-1 underwater autonomous robot.

This article is organized as follows: Section 2 summarises the state of the art concerning autonomy, resiliency and fault-tolerance in UUVs. Section 3 introduces the UX-1 robot used for a proof-of-concept and its redundant components, key for providing alternatives to the metacontrol. Section 4 presents metacontrol as a knowledge-based architecture to take advantage of self-information to be able to adapt to operational requirements. Here, the TOMASys system realization metamodel is presented as the framework whereupon the metacontroller is implemented. Section 5 addresses the concrete aspects of the adaptation experiment described in this article. It specifies the UX-1 subsystems that are subject to the metacontroller and used to reason about and reconfigure during operation. Section 6 describes the results of the metacontrol experiments with UX-1. Finally, Section 7 discusses benefits and limitations of the metacontroller as proposed and demonstrated. Future research lines are also identified. Section 8 presents concluding remarks.

## 2. State of the Art

The work described in this article addresses the need for autonomous operation of underwater robots. The following three sections address the core issues as stake in our work: the importance of a flexible approach for autonomy, the augmentation of resilience by system adaptation and the importance of self-X for autonomous robots.

### 2.1. Flexible Approach to Robust Autonomy in UUVs

**Figure 1:**
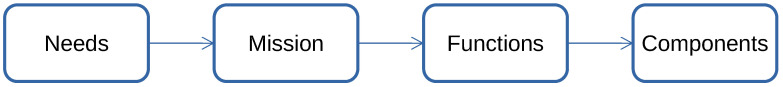
The system engineering flow from the needs of the final user to the concrete realization of the robot system as an aggregate of components.

The system engineering process [3] proceeds from the needs of the final user to the concrete realization of the system as an aggregate of components that are integrated and deployed in the operational environment (see Figure 1). This process applies also to the engineering of an UUV: needs are related to science, war or business and the final UUV components that compose the deployed system are the thrusters, cameras, comms, etc.

The common strategy to achieve system dependability is straightforward: identify likely disruptions—internal and external—and design, build and deploy system capability for handling them. One of the most common strategies to handle component fault disturbances is the use of component redundancies and a mechanism to use them (e.g., Triple Modular Redundancy (TMR), or the use of hot/warm spares). Concerning adaptation to external disruption, the common strategy is the set up of alternative system configurations and the active selection of them based on situational awareness mechanisms (for example using sliding mode controllers [4]).

All these methods suffer from the same drawback: they are ad-hoc solutions to very specific anticipated problems. They have three major issues: (1) they may not effectively use all the resources of the robot; (2) they may not ensure a correct operation, and (3) they do not generalize well (i.e., apply to unforeseen circumstances). The mapping situation→solution is too rigid. A more general and flexible approach to robust autonomy is in need.

### 2.2. Resilient Robotics

Resilience is the capability to recover from failures to maintain its operation [5]. It represents the action of returning the system to the point of balance for which it was designed. This term is often confused with robustness, which is the ability to withstand in presence of perturbations or damages [6]. Both characteristics are relevant for increasing robot autonomy, but they handle the problem differently. Whereas resilience constitutes an active adaptation to keep the robot functioning after a fault, robustness represents a preventive approach to avoid malfunctioning and keep operation in presence of disturbances. In this article, we present a strategy to increase systems resilience. We target an architectural reconfiguration to ensure mission fulfillment.

The use of resilience in machines and dynamical systems is not new. Sun et al. [7] presents an architecture of the resilient machine that includes a five-layer approach. First, it requires a redundancy structure, this includes modular redundancy and functional redundancy. The second-level structure is a management system to decide how the system configuration changes. The third level is a monitoring system to evaluate the system’s performance and the state of the components. Later, they define a system to train the elements to perform a new function. The last step is an actuation system to implement the new configuration.

The work presented in this article constitutes the validation of a general approach to applying resilience to a different type of system, in this case, the UX-1 robot. Metacontrol implements a resilient structure from a system-independent perspective which is then particularized for each application (see Section 4.1). Metacontrol uses the modular and functional redundancy to adapt to contingencies, acting as a structure-manager by using logical reasoners to evaluate if the system requires reconfiguration and select the best design alternative. An observer monitors each reconfigurable module of the system to evaluate its functioning. When reconfiguration is required, the metacontroller selects the most suitable functional alternative and implements them in the system. The novelty of our approach is the use of ontologies and logical reasoners to perform reconfiguration, as it constitutes a reusable top-layer for resiliency. The use of ontologies to store all the design and run-time information of the system is the tool selected for reusability (see Section 5.2).

The problem addressed in the UX-1 case is motion when a thruster is malfunctioning. This is similar to [8] in which a resilient self-repair control of aircraft rotors is implemented. Another solution for motion is provided by [9]. There, a resilient design is used to keep walking when one of the four legs is damaged. This approach uses self-models to generate alternative gaits to maintain locomotion. Similarly to this article, the metacontrol approach implemented here uses self-models to recover from damage. Another example of self-models usage is [10], which employs self-assembly and reconfiguration on modular robots. In this case, they use the function-context-behavior-principle-state-structure (FCBPSS) model [11], which is a semantic framework to define the system domain model. Our metamodel implementation focuses on a general system model framework to govern the reconfiguration.

### 2.3. Fault-Tolerance and Adaptation

System fault tolerance is a well-known engineering discipline developed in the domain of dependable systems [12,13,14]. However, to properly understand the issues dealt with in the work described in this article it is necessary to put them in the perspective of a full systems engineering life-cycle [15] as described in the previous section (see Figure 1).

The domain of robotics use the same class of fault detection and fault tolerance mechanisms [16] as other technical domains. For example Cui et al. [17] describe a self-adaptation framework to support fault tolerance in fielded mobile robots. Guo et al. [18] have produced a ROS-based software framework for fault-tolerant mobile robots. Software fault tolerance and adaptation is a well-developed domain and hence of major value in intelligent robotics. Concerning more physical aspects, for example, Sarkar et al. [19] describe a system capable of performing thruster force allocation of an UUV in faulty situations considering thruster redundancy and their potential saturation. Ni [20] and Ding et al. [21] describe sliding mode and Motion Prediction Control (MPC) controllers for ad-hoc fault tolerance in UUVs. The space probes domain has addressed similar issues [22].

Antonelli [23] makes a survey over existing fault-tolerant schemes for UUVs, where sensor and thruster failure both in software and hardware are successfully handled in operational conditions. The consideration on most of the Fault Tolerance (FT) schemes are thruster redundant vehicles with 6 degrees-of-freedom even after the fault. This is also the scheme followed by the experimental setup of this article.

Usual faults aligned with thruster unavailability are:

- Rotor failure. Zeroing the blade rotation so the thruster stops working. An example of this failed experiment can be found in [24].
- Thruster blocked. A solid body situated between the propeller blades to the thruster requires more current, as stated in [25]. In extreme cases, this can be solved by deactivating the blocked thruster.
- Flooded thruster. If water gets into a thruster, there is an electrical dispersion that may increase the blade rotation. Again this can trigger an unsafe level of force on the thrusters that may be handled by deactivating the flooded thruster. This problem is also addressed in [25].

In these cases, usually the action taken in a fault-tolerant approach consist in zeroing the reference signals to the faulty thruster. In practice, this consists of the zeroing of the column corresponding to the broken thruster from the thruster *configuration matrix*. However, in performing this operation it must be guaranteed that the resulting matrix satisfy some constraints: it must be *rectangular-enough* to allow the UUV control in all the 6 degrees-of-freedom. This is a mathematically complex problem that only specific set-ups such as the one reported in [26] have addressed. In this work, we provide some design-criteria to overcome this mathematical complexity. In our implementation, a further step has been taken as this matrix adapts to this zeroing action (see Section 5 and Equation (4)).

The use of system engineering and component-based architectures is not novel for reusable *self-adaptable* UUVs. As mentioned in [27], innovation, particularly in the marine robotic context, need not necessarily be developed from scratch but can be built on existing infrastructures. In this article, reusability is one of the main targets. The aim of our research is to explore a transversal technology in which some particularities are application dependent but there is a strong core of substantial ideas functional for a variety of systems. In this field, Gerasimou et al. [28] presents a predefined set of ocean surveillance missions, adaptation scenarios, and controller implementations which are integrated into the UNDERSEA tool, [29]. In [30], the uncertainty of self-adaptive systems is handled through requirement thresholds and component interactions. Further research on this field has been made in the RODIN project (Rigorous Open Development Environment for Complex Systems) with special emphasis on design techniques to develop software-based systems [31].

From the Sifakis [32] perspective, autonomy is characterized by knowledge handling and adaptation response. The objective is to maintain functionality rather than use certain techniques. In this field, Zhai et al. [33] presents a reasoner based on the Web Ontology Language (OWL) and the Semantic Web Rule Language (SWRL) to manage knowledge with underwater heterogeneous robot during cooperative missions.

In this work we adopt the TOMASys metamodel (see Section 4). TOMASys approach also uses OWL and SWRL reasoning for managing the use of heterogeneous components in an UUV. However, TOMASys takes a further step. Zhai et al. [33] uses the reasoner to define an information model through semantic queries. In our implementation, the information model is used as a diagnostic tool to trigger the adaptation and reconfiguration of the robot.

### 2.4. Self-X for Robots

The technology that we are exploring in this work can be described as Self-X [34]: using system reflection for any task. In the world of pure software systems this strategy is commonly named self-* or self-adaptive systems and its origins can be traced back to IBM Autonomic Computing initiative. However, most of these systems are just simple controllers for computing systems [35].

Using control loops to address run-time uncertainty is common trade in automatic control. A meta step beyond simple control is the use of reflective controllers: controllers that control themselves. The use of reflective closed control loops is not new, but a common, old strategy in the engineering of adaptive systems [36]. Adaptive controllers have been used for all kinds of systems—including software systems—to keep them aligned with run-time changes.

In the work described here we try to go one step further: the control loops use *deep knowledge* about (i) the controlled system—the UUV in our case—, (ii) its controller—a ROS-based robot controller—and (iii) their relation to perform run-time system reorganization based on a deep understanding of the mission↔system relation. To achieve this, the system must be aware of both the environment and itself (i.e., be *self-aware*).

## 3. The UX-1 Robot: A Flooded Mine Explorer

Underwater Robotic Vehicles (URV) are usually divided into two categories, Remotely Operated Underwater Vehicles (ROV) and Unmanned Underwater Vehicles (UUV). Although UUVs are more restricted in terms of operation and power consumption, they are more autonomous and maneuverable because of their control capabilities [37,38].

The UX-1 is an UUV developed to explore and map underground flooded mines. The aim is to provide geological, visual and spatial data of hazardous areas that currently are expensive and risky to explore. In Europe, there are around 30,000 closed mines with a considerable amount of mineral raw materials. Many of them were abandoned because of low commercial revenue or expensive and dangerous exploration [39]. The UX-1 robot is designed for different applications: open new exploration mines for raw materials, define more informed drilling plans for intricate tunnels and unknown topologies, improve geosciences understanding through new data, exploration of dangerous areas such as nuclear accidents or toxic spills, surveying unstable underwater environments after an earthquake, etc.

**Figure 2:**
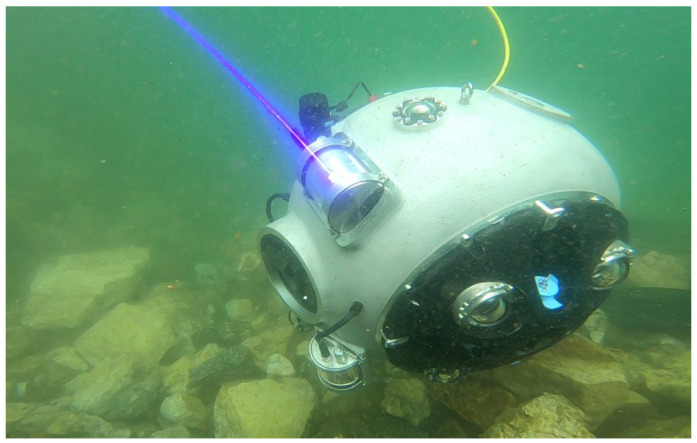
The UX-1 prototype during operation in Kaatiala Mine (Finland). Image credits: UNEXMIN, www.unexmin.eu, (Accesed: 30 December 2020).

The requirements imposed by these applications have ended in a spherical robot with a diameter of 0.5 m with high maneuverability capabilities. The UX-1 could not have any protruding elements that may collide with the intricate underwater deposits. For this reason, the propulsion manifold is integrated into the hull, an image of the UX-1 prototype can be found in Figure 2.

When a robot is in operation and suffers a severe disruption, the robot base configuration is not capable of handling it because the situation is outside the base set of design assumptions. In the case that the severe disruption comes from a system fault, the fault-tolerant mechanism takes control to recover the operational capability of the robot. The recovery process is automatic and *implicit* because the systems engineering design knowledge that links functions to components is generated, used, and lost at design time and is not available at run-time. Metacontrol provides a tool for run-time reasoning with a knowledge base which is based on that system design information. The UX-1 prototype is an ideal option for testing metacontrol and self-awareness capabilities for two reasons, it is a highly redundant system and operates in complex environments such as the flooded mines.

**Figure 3:**
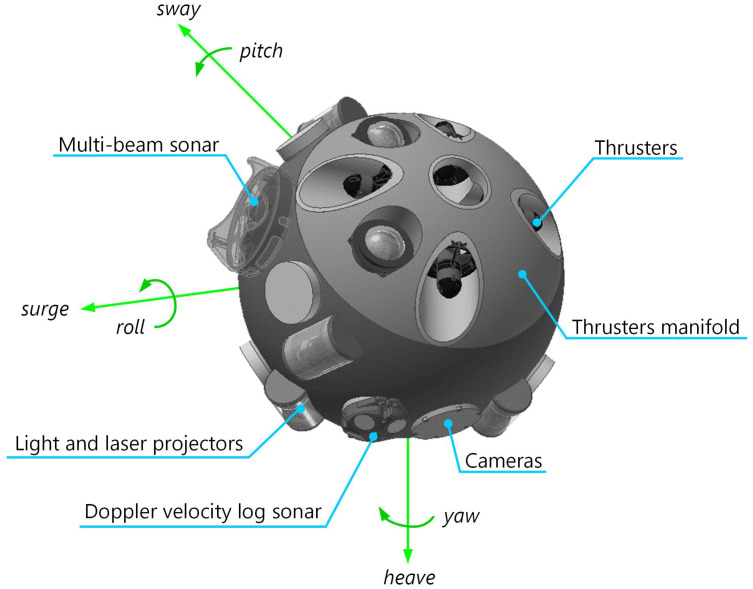
In blue, main components of the UX-1 robot for navigation; in green, description of motion coordinates according to Society of Naval Architects and Marine Engineers (SNAME).

For metacontrol testing purposes, we have focused on the motion system of the UX-1. The propulsion system is composed of eight thrusters (four per side, arranged crosswise). This results in five degrees of freedom: *surge*, *heave*, *yaw*, *roll* and *sway*; according to the Society of Naval Architects and Marine Engineers (SNAME), see Figure 3. Additionally, a pendulum allows the *pitch* rotation and an additional ballast for a faster heave motion.

**Figure 4:**
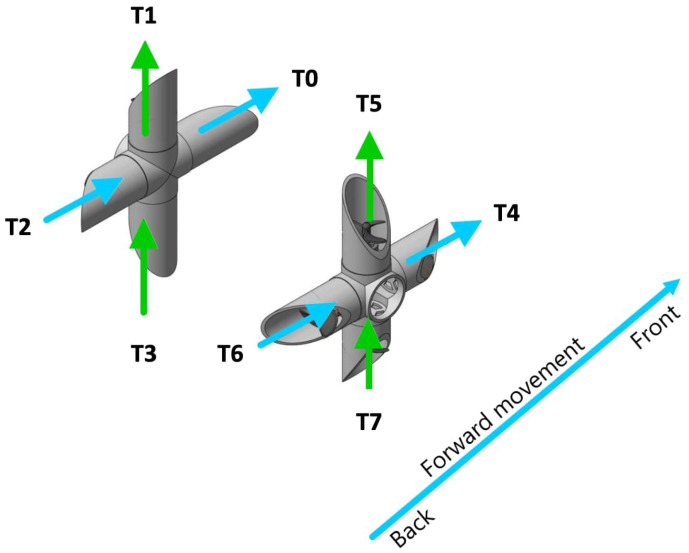
Thrusters allocation, the green arrows represent the thrusters implicated on heave movement ($T_{1}$, $T_{3}$, $T_{4}$ and $T_{7}$) and the blue ones, the thrusters responsible of forward movement ($T_{0}$, $T_{2}$, $T_{4}$ and $T_{6}$).

To test the metacontrol and self-awareness capabilities, only two degrees-of-freedom are taken into account: *surge* and *heave*. Surge is the linear longitudinal (front/back) motion in SNAME terms. Likewise, heave is the linear vertical (up/down) motion. The reason to use these directions is because they are the two main movements for travelling during an expedition. Furthermore, each movement uses a different set of thrusters so reconfiguration in presence of fault is enriched with adaptation to the motion direction. In Figure 4, thrusters in blue ($T_{0}$, $T_{2}$, $T_{4}$ and $T_{6}$) are responsible for surge whereas thrusters in green ($T_{1}$, $T_{3}$, $T_{4}$ and $T_{7}$) are responsible for heave. So if $T_{0}$ breaks during the shaft descend, no reconfiguration is needed until the start of the forward movement, as this thruster is not implicated in heave. With this approach, metacontrol ensures the use of maximum performance architectures when possible; further detail can be found in Section 5.

### UX-1 Mission Description

**Figure 5:**
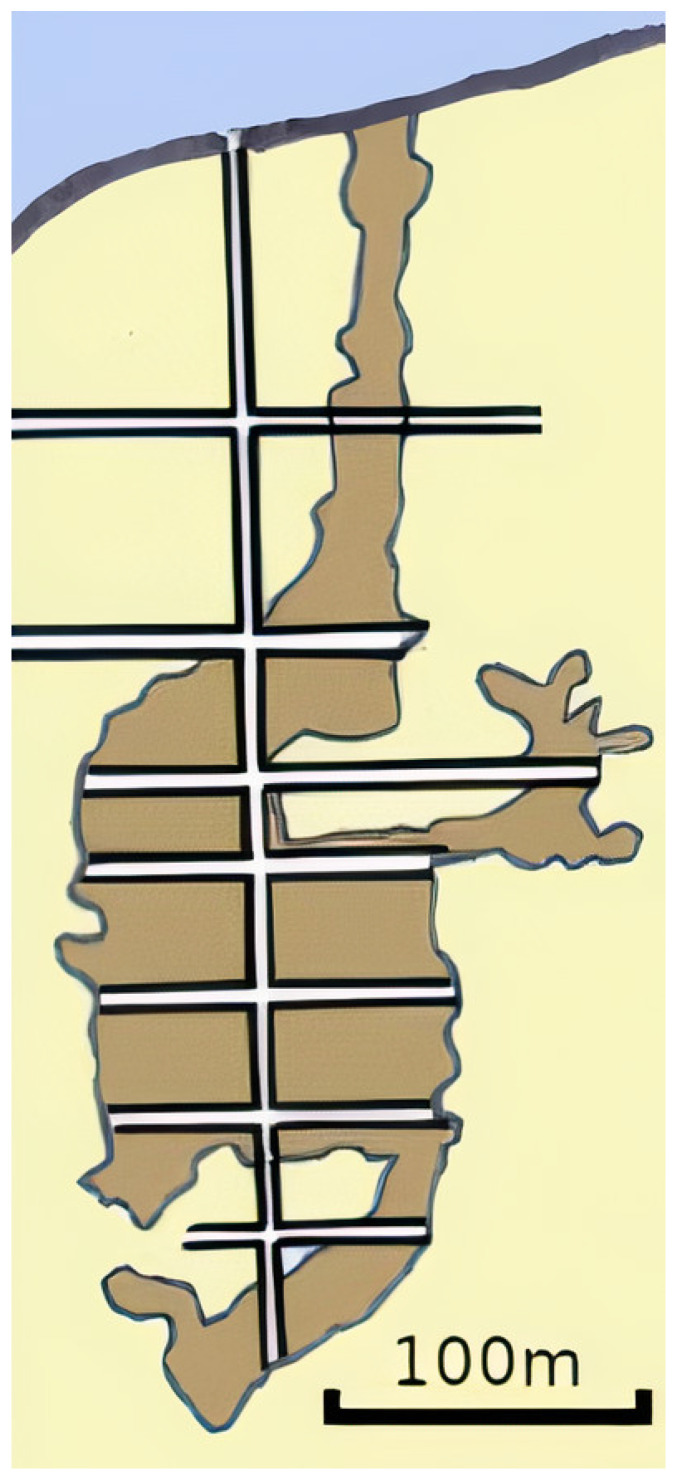
Simplified cross section of the Ecton mine in the UK.

The UX-1 robot is designed for the exploration of underground flooded mines. In this experimental setup, we focus on one possible scenario in a typical underground mine network, that is, a vertical shaft extending downwards connected to a perpendicular horizontal tunnel. This structure can be found in various mine workings, such as the mercury mine Idrija in Slovenia, the uranium mine Urgeirica in Portugal, and the Ecton copper mine in the UK, where the second, third and fourth field trials of the UNEXMIN project, respectively, took place, and represents the starting point of numerous mine sites: hence its interest. As an illustration, in Figure 5, a simplified cross-section of the Ecton mine is depicted.

In this proof-of-concept experiment, the mission consists of the following steps: descending a shaft from the deployment location of the submersible, i.e., the surface; entering and traversing a tunnel; and returning to the deployment location. To perform this type of mission, the submersible moves in four directions independently: downward, forward, backward, and upward. Movements in downward and upward directions are referred to as *heave* in the SNAME convention, whereas movements in forward and backward are referred to as *surge*.

## 4. A Metacontrol Architecture Using Self-Knowledge

Metacontrol is the instrument to pursue mission goals in the presence of disturbances with enhanced capabilities. This control loop is based on explicit models of the system. The solution designed in [40] and implemented here defines each controller as a domain and a metacontrol subsystems. The *domain subsystem* is the traditional controller, which does the sensing and acts on the plant. The *metacontrol subsystem* is the innovative approach, where the objective is to fulfill the system requirements. In this case, the plant to control is the domain subsystem. Hence, the metacontroller closes a loop on top of the regular control loop. However, to allow metacontrol, the domain subsystem must have some special features. First, it is required some redundancy to grant reconfiguration on the system. This can be reached within a variety of aspects: control laws, algorithms for behavior, structural components, etc. Furthermore, it has to allow monitorization so processes and elements have to report in real-time information about is operation. Lastly, the whole system must be designed in a way that reconfiguration is possible. This is the possibility of change, from parameter values to replacement, elimination, or declaration of components. Concerning the metacontroller, besides the monitorization of other subsystems, dynamical changes must be taken into account during reconfiguration.

In this case, the domain subsystem is in charge of navigation in nominal conditions. When a thruster-failure occurs, the metacontrol detects a deviation in the nominal conditions. Then, the robot cannot reach the mission with the nominal control laws. The metacontroller takes action through reconfiguration. To do so, a knowledge-base is used to specify the most suitable configuration according to different component failures and tasks. Then, a new set-point is defined to the domain controller according to the new configuration, so the system continues it operation.

### 4.1. TOMASys: Metacontrol Framework

The metacontroller used here is based on a metamodel called TOMASys (Teleological and Ontological Model of an Autonomous System) [40]. TOMASys is a theoretical framework aimed at reaching autonomy independently of the application and the system. This metamodel has a perspective both teleological and ontological. The teleological approach represents the incorporation of engineering-knowledge, that is, the intention and the purpose of the designers into the system model. Likewise, the ontological perspective represents the depiction of the structure and the behavior of the system.

As it is based on reaching an agent’s goals which are organized hierarchically in a teleological model. Self-awareness and adaptability are achieved by making use of general and specific knowledge through ontologies, organizing them in a model. The metamodel is exploited at run-time by the metacontrol subsystem to ensure the fulfillment of the system requirements. To do so, this metamodel is formal enough to be read by a machine. In other words, TOMASys makes a explicit representation of both the system structure and the function of its components, which are activated or deactivated according to the situation in response to the ontological reasoner.

TOMASys metamodel is inspired by component-based software in the definition of system structure and its functionality. The model elements are divided into two main groups. Static knowledge is stored in Functions and Function Designs. The Function element allows the definition of the abstract Objectives that the system must achieve. Function Designs are design alternatives to execute a Function.

The instantaneous state, by contrast, is captured with Objectives that define a hierarchy of the system requirements pursued at run-time, and Function Groundings, that specifies the run-time use of a Function Design. Components are also part of the instantaneous state, as this specifies the structural modules used at that instant.

**Figure 6:**
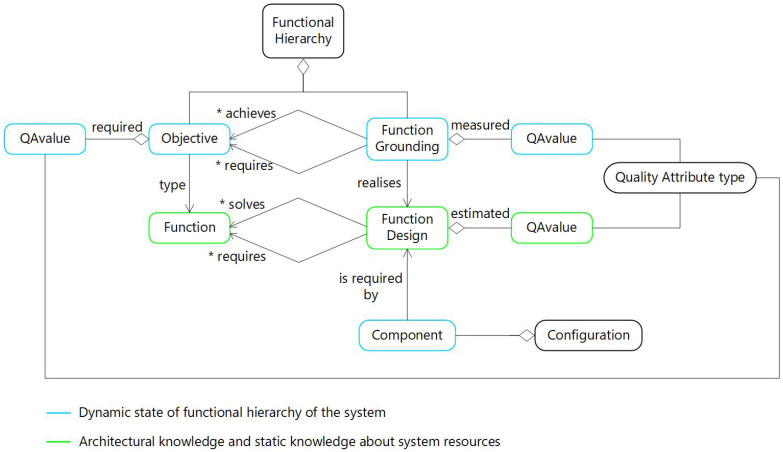
Main elements of Teleological and Ontological Model of an Autonomous System (TOMASys) metamodel, the * symbol represents the possible multiplicity in the destination relationship.

Quality Attributes are entities that affect both static and run-time knowledge. Quality Attributes are used to measure how the system fits the mission fulfillment. Each Objective has some requirements associated. Common Quality Attributes requirements are defined in terms of safety, energy consumption, performance, etc. Each Function Design has some estimated Quality Attribute Values to allow the selection of the best alternative according to the situation. Lastly, each Function Grounding has some measured Quality Attribute Values to reify the estimations of Function Design with run-time perceptions. An overview of the TOMASys metamodel is shown in Figure 6.

### 4.2. Ontological Reasoning and Metacontrol

TOMASys constitutes the TBox (assertion on concepts). The term *TBox* describes the terminological components of the knowledge-base in contrast to the *ABox* (assertion on individuals) that are the TBox-compliant statements that use the terminology. Therefore, TOMASys defines a formal, application-independent vocabulary to developers to facilitate reusability among different applications, particularly in hierarchical, component-based systems.

To make use of TOMASys metamodel, a knowledge base specific for the system and its mission is required. The ABox defines the specific individuals of the application in terms of TOMASys TBox. Conclusions extracted are not only relative to faults, but also about real-time performance and efficacy of involved components. With this information, the system will be able to adapt to keep operating after damage while reaching the best competence among its capabilities.

The ontology is composed of two files: The TOMASys TBox and the application-specific ABox, UX-1 navigation in this case. Both of them are written in OWL-DL language (Web Ontology Language—Descriptive Logic). Then, they are used at run-time by a DL reasoner to diagnose the system and compute the reconfiguration when it is necessary. The ontology upon this reasoning acts as the knowledge provider on which self-awareness is founded.

Functional diagnosis is done by asserting the information about components. The reasoner provides the inference of the status of the set of Objectives and Functions Groundings from the TOMASys metamodel. If the levels of performance and efficiency are under expectations, the metacontroller proposes the best reconfiguration with the resources available. To do so, specific rules design for the system must be combined with general TOMASys semantics. In recent works, the TOMASys framework has been implemented with an OWL ontology with SWRL rules [41]. In that article, the authors explore a theoretical solution to address adaptation to thruster’s status using the TOMASys metamodel. Building on those results, in this article we present a complete proof of concept to address thruster failure and optimize navigation in a realistic operating environment. The proof of concept consists of an operational implementation of the metacontrol solution for ROS systems, including automatic ontological reasoning and software reconfiguration, and its application and test in a realistic scenario, for which a simulation of the UX-1 underwater robot has been developed. This implementation has been created as part of the Metacontrol for ROS systems (MROS) project [42], and is available as an open-source library [43].

## 5. UX-1 Self-Model and Metacontrol

In this paper we present the application of the metacontrol architecture to the UX-1 robot, with the objective to improve navigation performance in the presence of thruster faults. In this case, force reallocation is required to keep the submersible operating. A naive way of dealing with a thruster failure is disabling the symmetric thruster, i.e., the thruster in the same position but on the opposite side of the robot: this initial approach has been presented in Milosevic et al. [44]. However, in this work we test a more sophisticated approach avoiding the deactivation of a proper-functioning thruster, thus not wasting the available resources. The metacontroller acts here as a modular tool in controlling the robot behavior, changing the system parameters in presence of faults to continue the operation in optimal conditions according to the available thrusters. No human intervention is required so these parameter changes need to be integrated into the robot.

### 5.1. UX-1 Ontological Model

In this application the metacontrol loop focus on thruster failure. As not all the thruster are used in all the movement, different navigation functions have been designed. For *surge*, thrusters ($T_{1}$, $T_{3}$, $T_{5}$, $T_{7}$) are used; whereas ($T_{0}$, $T_{2}$, $T_{4}$, $T_{6}$) are used for *heave*. The ontology captures this knowledge making use of the classes defined in TOMASys.

**Figure 7:**
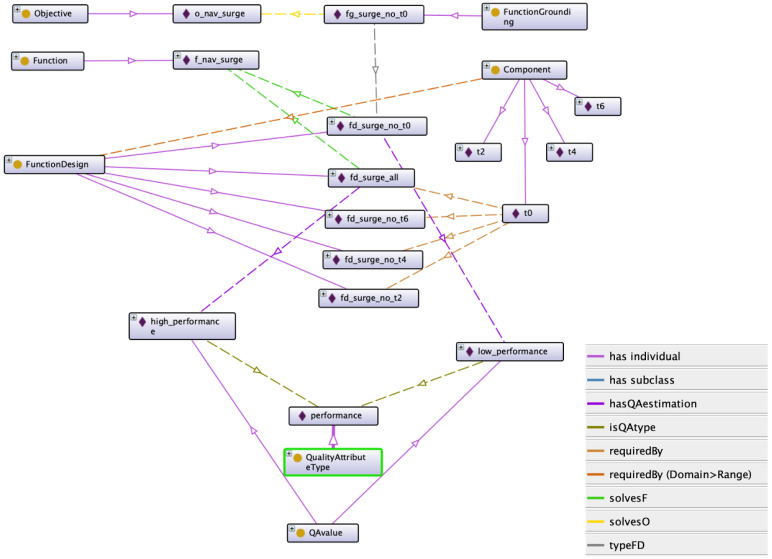
Main relationships affected by thruster $T_{0}$ contingency. The metacontroller uses the information in the knowledge-base partially represented here to take action. The Objective *o_nav_surge* is fulfilled by the Function Grounding *fg_surge_no_$t_{0}$*, this Function Grounding is a realization of the Function Design *fd_surge_no_$t_{0}$* which solves the Function *f_nav_surge*. This Function is required as is the one that solves the Objective. Components required to surge are thrusters $T_{0}$, $T_{2}$, $T_{4}$ and $T_{6}$. Among all the possible Functions Designs, the one that does not require the broken thruster, $T_{0}$, is the Function Grounding in use, *fg_surge_no_$t_{0}$*. Additionally, Quality Attributes relative to *performance* are assigned to each Function Design to prioritize the use of higher performance ones.

An overview of some relationships between classes and individuals is shown in Figure 7. The Objective and Function Grounding individuals are created at run-time. When the UX-1 is descending the shaft, the reasoner gets a diagnostic message of this movement and sets the Objective to *o_nav_heave*. When it starts going forward, this Objective is deleted and instantiates an *o_nav_surge* Objective. Each time a new Objective is defined, the reasoner searches the best function design available and grounds it creating a new Function Grounding individual. In this example with the malfunctioning thruster $T_{0}$, it is the *fg_surge_no_$t_{0}$*.

Regarding the static knowledge, the UX-1 implementation uses two Functions, *f_nav_surge* and *f_nav_heave*. Each of them has five Function Designs that are alternatives to complete the motion in that direction. For the surge movement, we have *fd_surge_all* to use when all surge thrusters are available and specific Function Designs when any of the surge thrusters are disabled, e.g., *fd_surge_no_$t_{0}$*.

The selection among Function Designs is done with Quality Attributes criteria. Different Quality Attribute Types can be defined, such as safety, energy, or reliability. In this case, *performance* is used. A reasonable assumption is that the navigation performance depends on the number of thrusters used, and we consider only two possible cases: normal operation using all thrusters, or having a faulty thruster and using the rest. Therefore, two Quality Attribute values are defined, *high performance* for all the function design that use all the thrusters and *low performance* to navigate without a thruster.

There are several relationships among classes; however, the main relationship in this application are *requiredBy* that defines which components (thrusters) are required in each Function Design and *typeF* that links an Objective to the type of Function it solves.

### 5.2. Ontological Reasoning for UX-1

One of the main challenges of metacontrol is the implementation of reasoning at run-time. The design criteria is incorporated into the robot to allow the most suitable adaptation according to the situation. In this application, the reasoning is based on SWRL rules using Pellet [45]. SWLR stands for Semantic Web Rule Language, based on OWL DL and OWL Lite sublanguages of the OWL. SWRL implements Horn-like rules to make assertions of an OWL knowledge-base. The assertions are made through a reasoner. The selection of Pellet reasoner is due to its availability and fast performance using SWRL rules. Pellet is an OWL2 DL reasoner open source, based on Java. It can be used with Owlready2 library which allows the integration of Ontologies with Python.

The ontological reasoner used for this application is based on TOMASys structure, so is application-independent as long as the UX-1 knowledge-base is organized in TOMASys terms. This reasoner has been used previously in a dual-arm mobile manipulator and in a mobile robot navigation [46] and now is used for controlling the navigation of an underwater robot, evidencing the transversal approach of metacontrol.

The metacontroller is based on the well established MAPE-K loop, constituted on a Monitor, Analyzer, Planer, and Executer supported by Knowledge [47]. The monitor stage is implemented with an *observer node*. In the UX-1 case, the observer uses ROS diagnostic messages to inform the reasoner about two types of events, the failure in one of the thrusters and the movement direction change, Table 1 collects the thrusters used for each movement direction according to Figure 4. e.g., if the robot is describing a surge motion and $T_{0}$ is not available, no reconfiguration action is needed. However, when the robot switch to heave, a reconfiguration action is required to take into account the disabled thruster.

The remaining stages of MAPE-K (Analyzer, Planer, and Executer) are implemented by the *reasoner node*. First, the reasoner loads the knowledge-base, this is the UX-1 ABox ontology and its TBox backbone, TOMASys. Then, the metacontroller creates a default Objective. In this case, as the experiment is targeted to traverse an L-shaped tunnel, this first Objective is to navigate in the heave direction. When the Objective is created, a Function Grounding is set to specify an initial robot configuration. In this case, the selected configuration is the use of the four heaving thrusters for maximum performance.

The nominal functioning of the reasoner is checking if the Objective is in error status, this is the Analysis phase of the reasoner. Two causes can set an Objective in error. First, when the observer notifies that one thruster is not working, the reasoner updates the knowledge-base. As a thruster is a component, its component status is set to false. Then, the Pellet reasoner makes the corresponding assertions according to the state through TOMASys SWRL rules. Table 2 shows the rules used in this case.

First, it checks if the component with a false status is used by the current implementation (Function Grounding). If the grounded function uses the disabled component, the Function Grounding status is in error, rule no. 1. Likewise, the error is propagated to the Objective through rule no. 2. Lastly, the Function Designs realisability changes according to the available components with rule no. 3. For instance, if $T_{0}$ is not working, the only available function design will be heave without $T_{0}$ and all surge designs. If the disabled thruster is not used in the current Function Grounding, the Function Design realisability is also set to false, as this result will be used when the objective change, e.g., the case while doing a heave movement, when switching to heave without $T_{0}$ the adaptation will use the realisability asserted in previous analysis loops.

When there is a change in the movement direction, the Objective must be reformulated. This is done by checking the link between Functions and Objectives, typeF as is shown in Figure 7. If the new direction does not match the Objective typeF relationship, the reasoner destroys the current Objective and creates one according to the new direction.

When an Objective is in error, the reconfiguration action is triggered, this is the Planner and Executer phase. The reconfiguration starts with the search for a new Function Grounding. The selection is made according to thruster availability, and performance-level to prioritize the use of all thrusters if possible. This is done first by checking the realisability and then the quality attribute values of performance linked to each available Function Design. The grounding of a new Function Design constitutes the reconfiguration that is realized in the robot by publishing a *reconfiguration message* in this case. The reconfiguration from the robot perspective is detailed in Section 5.3.

Therefore, this reasoning architecture supports three failure cases. First, if one thruster responsible for the motion in course fails, reconfiguration is triggered. Second, if one thruster not involved in the current motion fails, the status is stored to be used when the involved motion change. Third, failure of two thrusters, one responsible for each motion. In this case, the system selects a design adaptation for the motion direction in use as *surge* and *heave* directions are decoupled in motion. The case of two or more thrusters in one motion direction is not addressed in this implementation. In this case, the reasoner launches a message to inform that there is not *Function Designs* available.

### 5.3. Reconfiguration in UX-1

In our application, the reconfiguration available in the UX-1 is the selection of a force allocation matrix. Each Function Design in the UX-1 ontological model (Section 5.1) corresponds to a different force allocation matrix, and it depends on which thrusters are used according to the following dynamical model. The non-linear equation in (4) models the motion for an UUV using the motion representation vectors in (1)–(3).

(1) $$\nu ={\left[u,v,w,p,q,r\right]}^{T}$$

(2) $$\eta ={\left[x,y,z,\phi ,\theta ,\psi \right]}^{T}$$

(3) $$\tau ={\left[X,Y,Z,K,M,N\right]}^{T}$$

(4) $$M\dot{\nu }+C\left(\nu \right)\nu +D\left(\nu \right)\nu +g\left(\eta \right)=B\tau$$

where ν is the linear and angular velocity vector, η is the position and orientation vector, and τ is used to describe the forces and moments acting on the vehicle. The motion model matrix are ***M***, which is the system inertia matrix, C(ν), the Coriolis and Centripetal term matrix, D(ν), the total hydrodynamic damping matrix. g(ν) is the vector of hydrostatic forces and moments for the gravitational and buoyant forces acting on the vehicle and ***B*** is used as a mapping matrix for thruster configuration. Further detail of this model can be found in [48].

The action taken by the metacontroller is targeted to the thruster configuration matrix, *B*. This matrix is used to define how the thruster configuration affects the dynamics of the UX-1 robot. The UUV is actuated with eight thrusters allocated symmetrically on each side of the vehicle. *B* is a 6 × 8 matrix, the rows are the six DOF, {X,Y,Z,K,M,N}, and the columns correspond to each thruster, {$T_{0}$, …, $T_{7}$}.

Based on the dynamics of the system and the effect of the thrusters, the matrix *B* is defined in (5).

(5) $$B=\left[\begin{array}{cccccccc} 1 & 0 & -1 & 0 & 1 & 0 & -1 & 0\\ 1 & 1 & 1 & 1 & -1 & -1 & -1 & -1\\ 0 & 1 & 0 & -1 & 0 & 1 & 0 & -1\\ 0 & -l & 0 & l & 0 & l & 0 & -l\\ 0 & 0 & 0 & 0 & 0 & 0 & 0 & 0\\ l & 0 & -l & 0 & -l & 0 & l & 0 \end{array}\right]$$

with

(6) $$l=sin\left(\delta \right)\sqrt{\sigma_{1}^{2}+\sigma_{2}^{2}}$$

where $\sigma_{1}$ is the distance from the axis of the thrusters to the geometrical center of the UX-1, $\sigma_{2}$ is the distance from each thruster to the middle lateral point, and $\delta =arctan\frac{\sigma_{2}}{\sigma_{1}}$ is the rotation angle of the moments generated on the UUV.

After experimental tests, this matrix is adapted to force limitations and particularities in the final thruster disposition. The real *B* used when all thrusters are well-functioning is presented in Equation (7); this is the configuration used with function designs *fd_surge_all* and *fd_heave_all*. Note that this matrix is independent of the direction of movement. When surging, the system will use the information in the first row (*X*); whereas when heaving, it will use the third row (*Z*).

(7) $$B_{real}=\left[\begin{array}{cccccccc} 0.5 & 0 & -0.5 & 0 & 0.5 & 0 & -0.5 & 0\\ 0.25 & 0.25 & 0.25 & 0.25 & -0.25 & -0.25 & -0.25 & -0.25\\ 0 & 0.5 & 0 & -0.5 & 0 & 0.5 & 0 & -0.5\\ 0 & 0 & 0 & 0 & 0 & 0 & 0 & 0\\ 0 & 0 & 0 & 0 & 0 & 0 & 0 & 0\\ 0.25 & 0 & -0.25 & 0 & -0.25 & 0 & 0.25 & 0 \end{array}\right]$$

**Figure 8:**
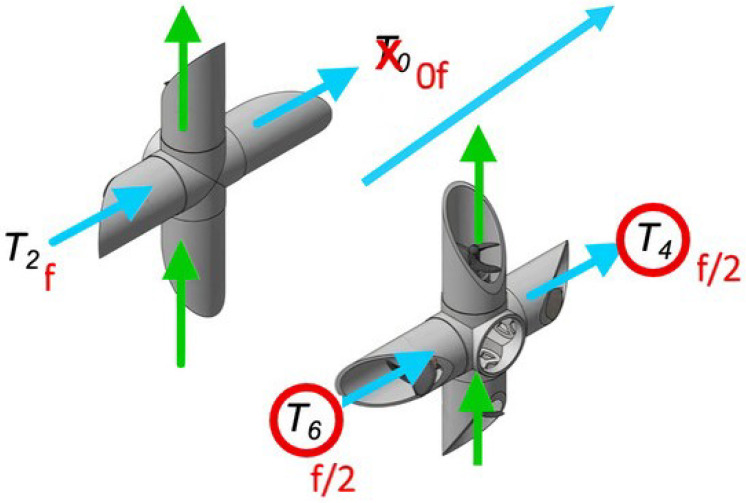
Force allocation in thrusters $T_{0}$, $T_{2}$, $T_{4}$, $T_{6}$ when surging.

When one thruster is disabled, e.g., $T_{0}$, its corresponding column is all set to zero. The force allocation depends on the movement direction, when one thruster is not functioning, the sum of forces made by one side needs to be equal to the other side to preserve symmetry. If $T_{0}$ is disabled, $T_{2}$ could double its force to compensate. However, for security reasons is preferable to preserve the nominal workload in $T_{2}$ and divide it by half in the other side, $T_{4}$ and $T_{6}$, see Figure 8.

The adaptation of the *B* optimal matrix in (7) to the case of thruster $T_{0}$ disabled while surging, result in the matrix (8).

(8) $$B_{noT0}=\left[\begin{array}{cccccccc} 0 & 0 & -0.5 & 0 & 0.25 & 0 & -0.25 & 0\\ 0 & 0.25 & 0.25 & 0.25 & -0.125 & -0.25 & -0.125 & -0.25\\ 0 & 0.5 & 0 & -0.5 & 0 & 0.5 & 0 & -0.5\\ 0 & 0 & 0 & 0 & 0 & 0 & 0 & 0\\ 0 & 0 & 0 & 0 & 0 & 0 & 0 & 0\\ 0 & 0 & -0.25 & 0 & -0.125 & 0 & 0.125 & 0 \end{array}\right]$$

The same force-conservative approach is taking when any thruster is disabled, adapting the force reallocation their motion contribution, surge in ($T_{0}$, $T_{2}$, $T_{4}$, $T_{6}$) and heave in ($T_{1}$, $T_{3}$, $T_{5}$, $T_{7}$).

When the metacontroller creates a Function Grounding entity from the selection of the most suitable Function Design, the reconfiguration is triggered. The reconfiguration consists of the adaptation of the *B* matrix according to the thruster availability. This matrix is stored in a comma-separated values (CSV) file. The name of the file corresponds to the Function Design reified by the Function Grounding.

The metacontroller publishes this matrix to a *reconfiguration* ROS topic for the low-level controllers, which will use this matrix to adapt the motion to the run-time situation, according to the motion model in (4).

## 6. Experimental Results

In order to validate the designed ontological reasoner and the metacontroller we have performed experiments following a software-in-the-loop approach, set through the combination of the Gazebo [49] simulator and a realistic model of the UX-1 robot. The position of the robot was acquired from Gazebo’s ground truth measurements, further disturbed with random Gaussian noise accumulated over time, to better mimic the positioning system of the real submersible based on noisy instant relative measurements and dead-reckoning. The controller used for the experiments is the Feedback Linearization (FL) controller developed for the UX-1 platform and presented and validated in detail in [48]. The experiments were performed on a 64-bit Ubuntu 16.04 PC with an Intel i7-6700 2.6 GHZ processor and 16 Gb of memory and using ROS Kinetic as middleware.

Three different experiments were performed, all of them reproducing the setting and steps explained in Section 3, but with different thrusters enabled/disabled. The first experiment, denoted as **I**, was performed with all thrusters working. The second experiment, denoted as **II**, was performed with a simulated failure of one of the thrusters in charge of the *heave* movement ($T_{0}$, $T_{2}$, $T_{4}$, $T_{6}$). Finally, the third experiment, denoted as **III**, was performed with a simulated failure of one of the thrusters in charge of the *surge* movement ($T_{1}$, $T_{3}$, $T_{5}$, or $T_{7}$).

The experiments were performed with analogous setting, and the commanded path was comprised of waypoints containing the desired location in space and the orientation of the robot, and were entered in the following format [x,y,z,ϕ,ψ], where the location variables *x*, *y*, and *z* follow the usual *North* (*x*)-*East* (*y*)-*Down* (*z*) convention for marine navigation, and ϕ and ψ represent, respectively, the pitch and yaw of the robot. The complete reference path was composed of the following 6 waypoints (x,y,z in meters, ϕ,ψ in degrees).

(9) A:(1.0,1.0,1.2,1.0,1.0),B:(1.2,1.0,1.2,1.0,1.0),C:(3.5,1.0,1.2,1.0,1.0),D:(1.5,1.0,1.2,1.0,1.0),E:(1.0,1.0,1.2,1.0,1.0),F:(1.0,1.0,0.5,1.0,1.0),.

In the experiment **II**, corresponding to the fault situation, said failure of the thruster $T_{0}$ was triggered during the *surge* movement while going away from the deployment location when the submersible reached the position x=1.7 m. In the experiment **III**, failure of the thruster $T_{1}$ was triggered during the *heave* movement while going away from the deployment location when the submersible reached the position z=0.8 m. The time elapsed between the trigger of the failure and the system reconfiguration, referred to as latency, in the experiment **II** was 1.81 s, and in the experiment **III** was 1.09 s. The measurement of these elapsed times was performed by comparing the timestamps of ROS messages, and therefore its precision depends on the timestamps’ quality. Since all the nodes were run on a single PC, we assume no considerable delay caused by ROS middleware. The latency is depicted in Table 3c.

**Figure 9:**
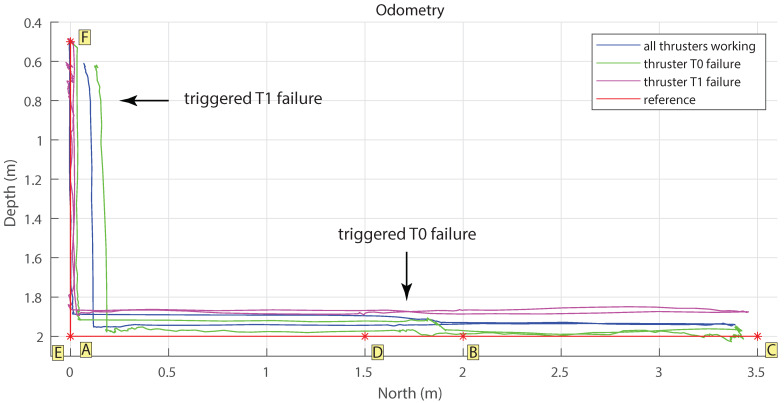
Odometry measurements during the thruster failure experiments. Yellow squares depict the reference waypoints.

The odometry measurement of each experiment is shown in Figure 9, as well as the reference waypoints depicted in alphabetical order corresponding to (9). The root-mean-square deviation (RMSD) of submersible’s position with respect to the ideal path, described by linking the commanded waypoints, is depicted in Table 3a. It can be noted that the RMSD for all experiments has similar values. This result is in line with the expectations and confirms the assumption of the redundancy of the UX-1 motion system, showing that the submersible can successfully perform the desired maneuvers (*surge* and *heave*, in this particular proof-of-concept) despite the failure of one of the thrusters.

**Figure 10:**
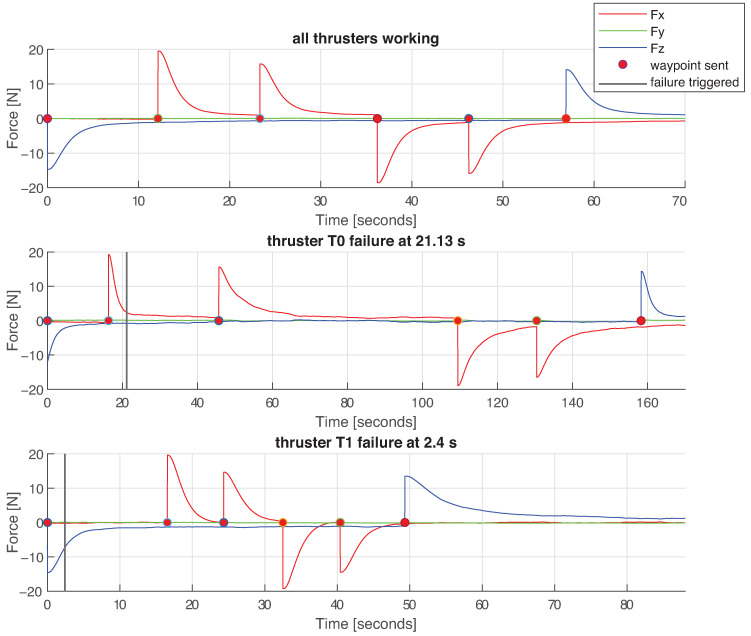
Force reference commands obtained by the FL controller. Uppermost figure represent the **I** test, middle the **II** and bottom the **III**. The time instance when each waypoint is sent is depicted with a red circle, and the moment of the thruster failure is depicted with a vertical black line.

**Figure 11:**
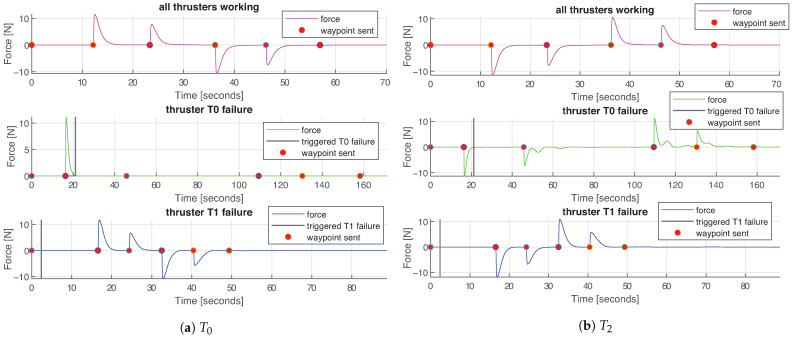

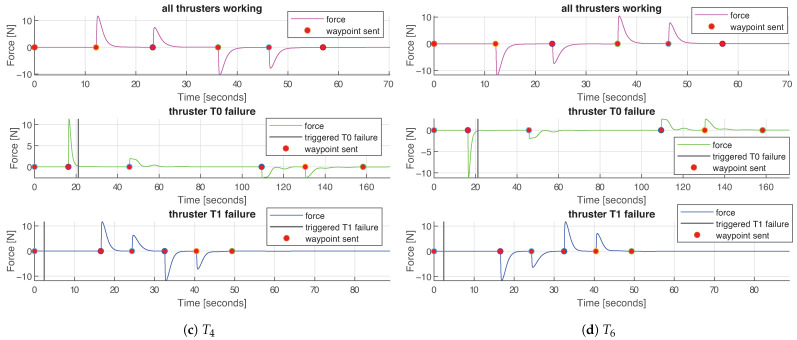
Forces produced by thrusters in charge for *surge* movement in all three experiments. Purple graphs represent the **I** test, green the **II** and blue the **III**. The time instance when each waypoint is sent is depicted with a red circle, and the moment of the thruster failure is depicted with a vertical black line.

**Figure 12:**
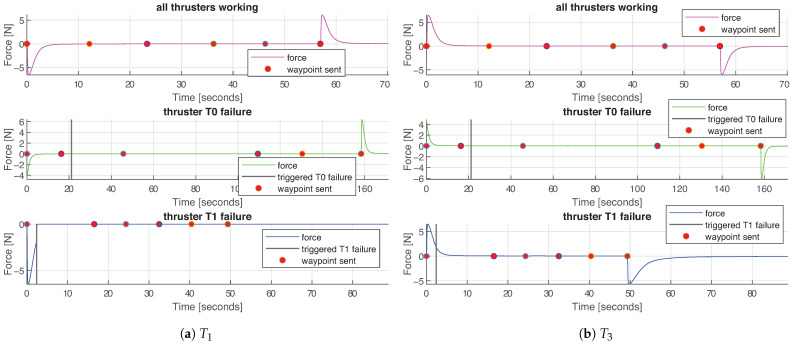

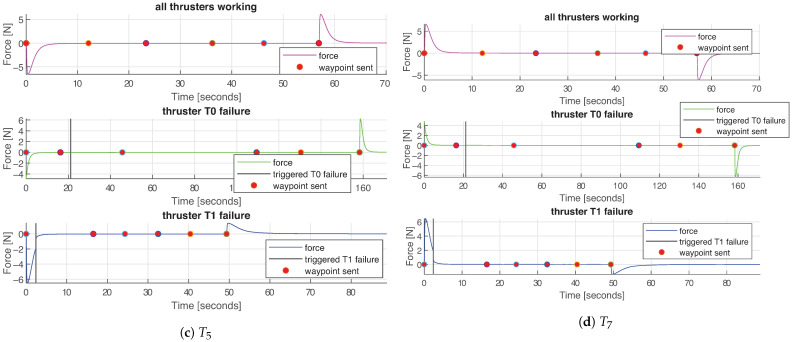
Forces produced by thrusters in charge for *heave* movement in all three experiments. Purple graphs represent the **I** test, green the **II** and blue the **III**. The time instance when each waypoint is sent is depicted with a red circle, and the moment of the thruster failure is depicted with a vertical black line.

Figure 10 depicts the force reference commands over time in all three experiments: that is, the force in each direction demanded to the thrusters by the controller, and Table 4a depicts mean values of each force reference. Figure 11 depicts the forces produced by each thruster in charge for *surge* movement, and Figure 12 shows the forces produces by each thruster in charge for *heave* movement. Table 4b summarized mean values of produced force per thruster.

It can be noted that the mean commanded force has almost identical values in all three tests: however, the forces actually produced by the thrusters are lower in the experiments **II** and **III**, compared to the experiment **I** in which all thrusters were working properly and with full power. The lower accomplished forces by the thrusters cause, as expected, the longer duration of the experiment (see Table 3b). The considerable difference in duration of the experiment **II** seems to be the result of the relative lengths of the vertical and horizontal sections of the experimental setting: the length of the reference path is longer in *North (x)* direction than in *Down (z)* (7 m versus 3 m); the longer distance travelled with reduced power in the thrusters in charge for that direction leads to the observed longer duration for completing the desired maneuver.

The green graphs in Figure 11, corresponding to the experiment **II**, visually depict the effect of the successfully performed run-time system adaptation of the submersible due to the thruster failure, explained in Section 5.3. The time instance when the thruster failure is triggered is shown with a vertical black line. After the waypoint **A** is reached and before the moment of the thruster failure, Function Design *fd_surge_all* is used, implying that all the thrusters are working properly and with full power. When the failure is simulated in the thruster $T_{0}$, the reconfiguration is triggered and the new Function Design is selected, that is, the one that does not require the malfunctioning thruster $T_{0}$, *fd_surge_no_$t_{0}$*. This Function Design implies the use of the opposite thruster on the same side of the hull, $T_{2}$, operating at the same power (Figure 11b), and the two thrusters on the other side of the hull, $T_{4}$ and $T_{6}$, operating at half power (Figure 11c,d). The reduction of the power by half can be perceived by comparison with the experiment **I** (graph in purple) when all the thrusters were working properly.

Analogously, the blue graphs in Figure 12, corresponding to the experiment **III**, illustrate the successfully performed run-time system adaptation due to the failure of thruster $T_{1}$.

Finally, the performed experiments, **II** and **III**, are compared to the initial naive approach consisting of disabling the symmetric thruster, i.e., the thruster in the same position but on the opposite side of the robot. Table 5 summarizes the numerical comparison between the experiments performed with the use of the proposed metacontroller and the experiments performed using the naive approach. Table 5a shows mean forces, Table 5b duration, and Table 5c latency of each experiment. It can be noted that the produced mean forces have similar values, however, higher values are obtained in the experiments with the metacontroller. This result is expected since the metacontroller avoids deactivation of a proper-functioning thruster; contrary to the naive approach. This result directly affects the tests’ duration, causing the longer duration of the experiments done with the naive approach. Finally, the latency of the system response to the thruster failure is compared and depicted in Table 5c. Both experiments have similar values, however, slightly shorter latency is obtained with the naive approach due to the usage of fewer ROS nodes.

These experiments demonstrate the viability of model-based metacontrol for reconfiguring the deployed system. This approach provides benefits that manifest both in (a) the mission fulfillment (in this case, shorter duration of the experiment in comparison with the most basic contingency handling, i.e., disabling the symmetrical thruster of the one malfunctioning) and (b) the generality of the systems engineering process. Reduction of engineering time is a clear advantage of model-based reasoners; however, from the point of adaptation response time, it is possible that most fault-handling results for a specific UUV in the literature will have better metric values (RMSD and/or latency) than ours, as they are designed specifically for a robot and its concrete operation.

The suitability of using metacontrol in terms of fault handling depends on the usually soft real-time bounding relation between measured adaptation time and mission requirements. When a fault is detected, the observer advises the metacontroller which changes the status of the component involved and asserts the reconfiguration required. Once the reconfiguration is selected, as the action is the selection of the thruster configuration matrix, so its usage is practically immediate. The bottleneck in the use of the metacontrol is the reasoning time for asserting the reconfigured system status and choosing the best design alternative depending on the objective and the contingency. In this implementation, we have used the Pellet reasoner, which offered a good trade-off in software availability and temporal performance [50]. In further work, the reasoning subsystem implementation may be improved using alternative reasoners and logic handling algorithms to reduce the time needed to reason about the fault. This will positively impact the adaptation time, decreasing the latency perceived by the system at the mission level.

Nevertheless, the value and strength of the model-based metacontrol approach are best seen from the systems engineering viewpoint, because it provides an application-independent, reusable adaptation engine that can be used to fast-implement a system-tailored module that can be deployed over any extant system with minimal additional instrumentation.

### Advantages of Using a General Approach

One may wonder why to use such a complex architecture for just selecting among different design alternatives. We could have used a number of simple ad-hoc *if* statements in the control code to adapt the robot behavior. However, the decoupling of reasoning assets from programming code allows not only the reusability of the metacontrol elements but also the reduction of the system and development complexity. This is a matter of separation of concerns that becomes critical when the complexity of the target system increases.

As of today, *if-else* hard-coded statements deeply embedded in ROS code are the commonly used mechanism to handle robot controller reconfiguration issues. But, as is well known in robust programming, handling emergent errors in deeply layered software is a tricky issue for programmers; especially if the code is complex [51,52] or has stringent requirements. In our case, as two system aspects are taken into consideration—motion direction and thruster failure—the complexity of the error handling problem directly maps to the number of *if-else* statements, since the addition of a new rule would require checking all the existing ones. In general, its estimated complexity is $n^{m}$ if we assume *m* aspects and an equal number *n* of variations per aspect. In our UX-1 case, the complexity can be estimated simply as 20, assuming 10 possible thruster configurations and 2 motion directions. Interestingly, in our ontology-based solution a formal vocabulary (our TBox), a set of general rules, and minimal module instrumentation—easily realizable using ROS nodes life-cycle resources—are all that is necessary to define and handle the error handling problem, instead of using a set of nested, pervasive *if-else* hard-coded statements. This way, once the developer is familiar with the language, adding a new variation in one of the aspects only requires the addition of the corresponding individuals and relationships in the ABox. The error handling system programming complexity is thus independent of the number of variants and depends only on the TOMASys vocabulary and the number and complexity of the rules in our ontology, which being Horn clauses have the same complexity than *if-else* statements. In the UX-1 case, the ontology contains 13 rules that are application-independent. For a simple system as is the case of our UX-1 thruster experiment, this difference maybe not very high; but for real robots, with tens of critical components, the difference may be substantial. This affects not only the system development time but also its quality and understandability; two critical factors that impinge on software reliability, maintainability, and extensibility.

One of the main advantages of using ontologies, particularly the TOMASys framework, is the usage of logic rules with a one to one mapping relationship. Most of the rules propagate the status of a Component to other entities such as the Objective or the Function Grounding. So, general actions can be taken depending on the entity affected.

When applying this technology in other applications it is possible that some system- or application-specific rules shall be added to the ABox to address new aspects. This, if properly done, shall produce an extension of system self-awareness knowledge because TOMASys, given its general systems foundation, provides a solid theoretical basis to ground and structure the knowledge growth process.

## 7. Discussion

In this work we address *self-X* concepts in robots; particularly, the use of these concepts in meta-control to close the control loop of the system at the mission level. This theoretical approach is validated in the UX-1 robot, an UUV used for floated mines exploration. The metacontrol here is targeted to ensure motion reliability in presence of thruster-failure. All the thrusters of the robot are used to ensure the movement in two directions: heave and surge. With the addition of an external control loop, when a thruster fails, no extra thruster is disabled for symmetrical force allocation. Therefore, the metacontrol approach augments the faulty-performance without human intervention.

### 7.1. Assessment of Present State

This application constitutes a proof-of-concept of the benefits that self-awareness and metacontrol provide to autonomous systems. TOMASys is a general framework, application-independent that is adapted to the particular system with an adequate ontology for reasoning. To use the TOMASys Architecture, a component instantiation is required with the particular elements that will be considered to metacontrol.

With these elements, we perform a run-time functional adaptation of the robot. The metacontrol provides a sub-system for recovery from component faults. This proof-of-concept has been conceived to maintain motion in presence of dysfunctional thrusters.

Moreover, the main limitation is imposed by the design constraints. We have a set of pre-defined function designs, these are design alternatives, and we select them depending on the run-time situation. The creation of new alternatives or the usage of available components for other tasks is a compelling research line to expand the metacontrol capabilities.

From the metacontrol encoded knowledge-base perspective, ontologies also have some limitations. In TOMASys, SWRL rules are used to make assertions about the UX-1, particularly the status of the thrusters, the objective, and the function grounding. SWRL is based on Horn rules, so it uses monotonic logic. This means that when a formula is added, the set of consequences is never reduced. As status are changeable values that evolve according to the run-time situation, this may be a problem. This problem has been solved through the metacontroller. The metacontroller handles this problem setting the Objective status and the Function Grounding status to *none* when an error is solved, so there is not overlapping status. This status change is not optimal as it is hard-coded in the metacontroller. Therefore, the ontology is used as an error-propagation system. When the observer detects a thruster malfunctioning or a change in motion direction, these observations are propagated to the Objective through SWRL rules in the ontology.

### 7.2. Future Work

Currently, we are working on reconfiguration representation and an evolving grammar definition to apply genetic algorithms to explore the best reconfiguration alternatives. Metrics on *optimality* and the cost of reconfiguration need to be addressed. Then, we plan to explore not only reconfiguration in hardware but also in software. In this work, software reconfiguration is limited to the force allocation matrix when any of the thruster is not available. Further work aims to adapt existing code to the operation conditions, changing and learning in real-time as a *live* system. In this case, reconfiguration conflicts will need to be addressed. Problems such as changing dynamics or merging information during the transition of states need to be taken into account.

The ontology implemented here was developed for this application but it is the ABox supported by the TBox for general autonomous robots, TOMASys. We plan to expand the ontology with ontological standards in robotics such as CORA (IEEE 1872-2015) [53]. Besides, the representation limitations of the ontology presented in Section 7.1 need to be addressed.

In this work, we have focused on keep motion. But motion itself does not usually define a real-world mission. Metacontrol must handle multiple goals organized in a hierarchy in order to achieve more ambitious missions such as localization of specific minerals in flooded mines, and also perform mining operations.

## 8. Concluding Remarks

In conclusion, we propose a generic, architectural, knowledge-based strategy to augment the autonomy levels of systems and, especially, of autonomous, unattended robots. Just as humans are able to evaluate the situation and predict changes to accommodate incoming disruptions, robots need self-diagnosis and self-adaptation to operate autonomously in the real world.

In a hazardous environment such as the underwater mine where the UX-1 robot operates, it is critical to have a trustworthy autonomous system because the possibility of human intervention is very limited. Using this novel architectural approach we have been able to increase the UX-1 UUV resilience and hence its reliability by endowing the propulsion system with a capability for adaptation to its own thrusters faulty state.

The metacontrol framework used here is general. It is domain and application independent. The use of a general systems metamodel, OWL ontologies and SWRL rules allows the reasoning in terms of hierarchical components and its attributes to control the different objectives instantiated on the model. We have applied these techniques to the UX-1 autonomous underwater robot proving its effectiveness and particularizing its use. Pending work remains, however, and interesting research questions have arisen in this projects which will be explored in future works such as the dynamic generation of reconfiguration designs at run-time, the ontological expansion to avoid SWRL limitations or the handling of multiple hierarchical goals.

Future work will therefore focus on more complex reconfiguration scenario, both of software and hardware. In particular, the robots being designed in two follow-up projects, UNEXUP (https://unexup.eu, [Accesed: 30 December 2020]) and ROBOMINERS (https://robominers.eu, [Accesed: 30 December 2020]) will build on the research presented in this paper. While in the former the same goals of advanced autonomy are pursued, in ROBOMINERS we are also addressing morphological reconfiguration on-the-job of a modular robot for the purpose of increased resilience.

## Figures and Tables

**Table 1:** UX-1 optimal thrusters used according to the movement direction, if one thruster is disabled the system must adapt to use the remaining thrusters in the column.

Surge	Heave
$T_{0}$	$T_{1}$
$T_{2}$	$T_{3}$
$T_{4}$	$T_{5}$
$T_{6}$	$T_{7}$

**Table 2:** Semantic Web Rule Language (SWRL) rules for UX-1 TOMASys implementation. The first one sets the Function Grounding in error if it uses a faulty Component, the second one sets the Objective in error if the Function Grounding is in error and the third one marks as unreachable the Function Designs that require unavailable Components.

***Rule no.1*** *tomasys:Component(?c) ⌃tomasys:c_status(?c, false) ⌃ux:requiredBy(?c, ?fd) ⌃tomasys:typeFD(?fg, ?fd) ⌃tomasys:FunctionGrounding(?fg) -> tomasys:fg_status(?fg, INTERNAL_ERROR)*
If a *Component* has a *Component status* in false (in error), and that component is *required by* a Function Design with the same *type* as the Function Grounding in use, then that *Function Grounding status* is set as *INTERNAL ERROR*.
***Rule no.2*** *tomasys:FunctionGrounding(?fg) ⌃tomasys:fg_status(?fg, INTERNAL_ERROR) ⌃tomasys:solvesO(?fg, ?o) ⌃tomasys:Objective(?o) -> tomasys:o_status(?o, INTERNAL_ERROR)*
If a *Function Grounding* has a *Function Grounding status* in *INTERNAL ERROR*, and that Function Grounding *solves* an *Objective*, then that *Objective status* is set as *INTERNAL ERROR*.
***Rule no.3*** *tomasys:Component(?c) ⌃tomasys:c_status(?c, false) ⌃ux:requiredBy(?c, ?fd) -> tomasys:fd_realisability(?fd, false)*
If a *Component* has a *Component status* in false (in error), and that component is *required by* a Function Design then that *Function Design realisability* is set to *false*.

**Table 3:** Comparison between tests. **I** represent the test with all thrusters working. **II** represent the test with the failure of $T_{0}$. **III** represent the test with the failure of $T_{1}$.

(**a**) The root-mean-square deviation (RMSD) of the vehicle position with respect to the reference trajectory		
**Test**	**RMSD**	**Units**
I	0.12	[m]
II	0.19	[m]
III	0.23	[m]
(**b**) Duration of each test		
**Test**	**Duration**	**Units**
I	70.2	[s]
II	168.7	[s]
III	88.3	[s]
(**c**) Latency between the thruster failure trigger and the system response		
**Test**	**Latency**	**Units**
II	1.81	[s]
III	1.09	[s]

**Table 4:** Mean force summary.

(**a**) Mean force reference commands obtained by the FL controller									
	**I**	**II**	**III**						
**Metrics**	**Value**			**Units**					
Mean Fx	2.93	3.01	2.72	[N]					
Mean Fz	1.73	1.72	1.92	[N]					
(**b**) Mean force produced by each thruster in each experiment									
	$T_{0}$	$T_{1}$	$T_{2}$	$T_{3}$	$T_{4}$	$T_{5}$	$T_{6}$	$T_{7}$	**Units**
**I**	0.82	0.82	0.32	0.32	0.82	0.82	0.32	0.32	[N]
**II**	0.09	0.29	0.13	0.13	0.29	0.5	0.13	0.13	[N]
**III**	0.59	0.59	0.1	0.17	0.59	0.59	0.39	0.17	[N]

**Table 5:** Numerical comparison of the experiments performed with and without the metacontroller (MC). **II** represent the test with the failure of $T_{0}$. **III** represent the test with the failure of $T_{1}$.

(**a**) Mean force			
	**II**	**III**	
**Test**	**Mean F**		**Unit**
With MC	0.21	0.4	[N]
Without MC	0.16	0.29	[N]
(**b**) Duration of the test			
	**II**	**III**	
**Test**	**Duration**		**Unit**
With MC	168.7	88.3	[s]
Without MC	203.2	113.1	[s]
(**c**) Latency between the thruster failure trigger and the system response			
	**II**	**III**	
**Test**	**Latency**		**Unit**
With MC	1.81	1.09	[s]
Without MC	0.91	0.72	[s]

## Data Availability

During the study, an open-source library has been created as part of the Metacontrol for ROS systems (MROS) project, https://robmosys.eu/mros, [Accesed: 30 December 2020], the tool is available at https://github.com/MROS-RobMoSys-ITP/mros_ontology, [Accesed: 30 December 2020].
